# Complete Genome Sequences of 11 Type Species from the *Thermococcus* Genus of Hyperthermophilic and Piezophilic Archaea

**DOI:** 10.1128/genomeA.00037-18

**Published:** 2018-02-22

**Authors:** Philippe M. Oger

**Affiliations:** a CNRS UMR5240, INSA Lyon, Université de Lyon, Villeurbanne, France

## Abstract

We report here the genome sequences of the type strains of the species *Thermococcus barossii*, *T. celer*, *T. chitonophagus*, *T. gorgonarius*, *T. pacificus*, *T. peptonophilus*, *T. profundus*, *T. radiotolerans*, *T. siculi*, and *T. thioreducens*, as well as the prototype of a possible type strain of a novel *Thermococcus* species, strain P6.

## GENOME ANNOUNCEMENT

*Thermococcus celer* strains were isolated from surface hydrothermal vent systems in the Mediterranean (1). *Thermococcus* sp. strain P6 was isolated in 1997 by M. Thomm (University of Kiel, Germany) from a hydrothermal site off Palaeochori Bay (Milos, Aegean Sea, Greece). *T. thioreducens* OGL-20P was isolated from the Rainbow Hydrothermal Vent System on the Mid-Atlantic Ridge, 2,600 m below the ocean surface (2). All other strains were isolated from hydrothermal vent systems in the Pacific Ocean (3–9) and exhibit very similar growth characteristics, with optimal conditions for growth ranging from 83 to 88°C, 5.8 to 7 pH, and 2 to 3% salinity.

DNA was extracted from cells grown in *Thermococcales* rich medium to late exponential phase. Whole-genome shotgun sequencing was carried out by a combination of IonTorrent PGM (800-bp fragment library, 318 chip, HiQ chemistry) and Illumina HiSeq runs (500-bp library, 100-bp paired-end reads), which generated at least 583,000 reads with a mean length of 389 bp (IonTorrent) and 13 million paired-end 100-bp reads after quality checking with FastQC (http://www.bioinformatics.babraham.ac.uk/projects) and trimming with Trimmomatic (10) for each species. The IonTorrent data were assembled *de novo* with Newbler version 2.8 (454 Life Sciences). The paired-end Illumina data were assembled *de novo* with Velvet (11). The pooled IonTorrent and Illumina data were assembled *de novo* with MIRA (12) and Newbler. The hybrid data assembly yielded between 1 and 6 contigs, which were connected by PCR.

Chromosome sizes range from 1.52 Mb in *Thermococcus* sp. strain P6 to 2.07 Mb in *T. thioreducens* OGL-20P, which are similar to known genomes from this family. Only two strains, *T. peptonophilus* OG-1 and *T. profundus* DT 5432, harbor an extrachromosomal element. The *T. profundus* DT 5432 plasmid belongs to the pTBMP1 family of plasmids described in the piezophilic archaeon *T. barophilus* (13). With the exception of *T. chitonophagus* GC74, the GC contents range from 51.7 to 56%, which are similar to previously sequenced *Thermococcales* strains. All genomes harbor 1 copy of the 16S-23S operon, 2 copies of the 5S operons, and 45 to 48 tRNAs, as well as large clustered regularly interspaced short palindromic repeat (CRISPR) loci associated with *cas* genes (*cas*1, *cas*2, *cas*3, *cas*4, *cas*5, *cas*6, and *csm*2), indicating multiple contacts with mobile elements of viral or plasmidic origin. Sinteny analysis confirms the plasticity of *Thermococcales* genomes. The gene content is consistent with an anaerobic and heterotrophic lifestyle. The genome of *T. barossii* SHCK-94 encodes the capacity for hydrogenogenic growth on carbon monoxide, as identified previously in *T. onnurienus* and *T. barophilus*.

The genome sequence of *T. peptonophilus* OG-1 (GC%, core genome phylogeny) confirms previous reports that it may have been incorrectly placed in the *Thermococcus* genus and should instead be placed in the *Pyrococcus* genus. Strain P6 is most closely related to *T. celer* strain Vu 13 (1), as indicated by *in silico* DNA-DNA hybridization of their genomes (predicted value, 28.90% using the genome-to-genome distance calculator GGDC version 2.0 [14]). This genomic distance suggests that strain P6 defines a novel species inside the *Thermococcus* genus.

### Accession number(s).

This whole-genome shotgun project has been deposited in GenBank under the accession numbers CP014750, CP014751, CP014854, CP014855, CP014862, CP014863, CP015101 to CP015106, and CP015193.
